# Qualitative exploration of uterine cancer care for lesbian, gay, bisexual, trans and queer (LGBTQ+) patients in the UK: shifting from equality to equity

**DOI:** 10.1136/bmjopen-2024-084720

**Published:** 2024-08-02

**Authors:** Nessa Millet, Rebecca Barnes, Natalie Darko, Esther Moss

**Affiliations:** 1 College of Life Sciences, University of Leicester, Leicester, UK; 2 Department of Medical Psychology, Amsterdam UMC Location VUmc, Amsterdam, The Netherlands; 3 Department of Population Health Sciences, University of Leicester, Leicester, UK; 4 NIHR Leicester Biomedical Research Centre, Leicester, East Midlands, UK; 5 University Hospitals of Leicester NHS Trust, Leicester, UK

**Keywords:** GYNAECOLOGY, Gynaecological oncology, Sexual and Gender Minorities, QUALITATIVE RESEARCH

## Abstract

**Abstract:**

**Objective:**

Patients identifying as lesbian, gay, bisexual, transgender and/or queer/questioning (LGBTQ+) report significant disparities in cancer care and are disproportionally affected by a cancer diagnosis on a number of health-related indicators. This study aimed to explore uterine cancer (UC) care from the perspectives of LGBTQ+ patients and stakeholders, to identify this population’s care needs, which have been underprioritised thus far.

**Methods and analysis:**

Qualitative interview data were collected from three cohorts of participants: LGBTQ+ UC patients, partners of UC patients and stakeholders who provide advocacy and/or support within the UC care pathway, including healthcare professionals (HCPs). Semi-structured qualitative interviews were conducted and data were analysed using inductive reflexive thematic analysis.

**Results:**

Fifteen participants (three patients, one partner, eight HCPs and three cancer support charity representatives) were recruited. Data analysis identified themes which represented participants’ reflections on the relevance and opportunities for identity disclosure during the diagnostic pathway; feelings and implications of not fitting into the gynaecological cancer environment and, opportunities and challenges surrounding HCP education, and protocolled sexual and gender identity data collection.

**Conclusion:**

UC patients who identify as LGBTQ+ have specific care needs and considerations, particularly related to transvaginal procedures and survivorship. Opportunities for disclosure of patients’ LGBTQ+ identity during the UC care pathway are essential for these needs to be recognised. Despite this, there are conflicting agendas between HCPs and stakeholders on the best approach to integrate disclosure processes. The current findings highlight the need for public health agendas and clinical services to address the needs of LGBTQ+ UC patients.

**STRENGTHS AND LIMITATIONS OF THIS STUDY**

- Incorporating multistakeholder perspectives from uterine cancer (UC) patients who identify as lesbian and trans masculine, a patient’s partner, healthcare professionals working in UC and charity workers offered a holistic examination of UC care for lesbian, gay, bisexual, transgender and/or queer/questioning (LGBTQ+) patients.
- Given LGBTQ+ UC patients’ marginalised identity within gynaecological care, qualitative methods enabled in-depth exploration of nuance in participant's experiences.
- The limited visibility of LGBTQ+ UC patients and lack of routine collection of sexual orientation and gender identity data in the National Health Service contributed to a small patient sample, influencing the ability to generalise to the wider LGBTQ+ UC patient population.
- Future research should prioritise the prior establishment of a patient and public involvement group to enable tailoring of research questions, to potentially facilitate recruitment and to sense-check findings.

## Introduction

 In the 2021 England and Wales national population census, 3.2% identified as lesbian, gay or bisexual, equating to over 1 537 000 individuals, and a further 0.5% of people identified as transgender;^1^ these figures are likely an underestimate.^2^ There is a growing agenda for cancer care to inclusively serve the needs of lesbian, gay, bisexual, transgender and/or queer/questioning (LGBTQ+) patients^3 4^ who reportedly face additional barriers to having their care needs met,^5^ experience unmet psychosocial care and support needs^3^ and are at risk of being disadvantaged due to mistrust and structural stigma.^6^ Additionally, LGBTQ+ cancer patients have reported being disproportionately affected by their diagnosis in terms of health-related quality of life, psychological distress, sexual well-being^7 8^ and a lack of social and familial support.^3^

Gynaecological care has been identified as a particularly challenging issue,^9^ with patients reporting concerns that disclosing their LGBTQ+ identity will jeopardise their care.^10^ Healthcare professionals (HCPs), clinicians and clinical nurse specialists (CNSs) play an important role in shaping the cancer care environment for patients^11^, which has the potential to be positive through a holistic care approach which demonstrates cultural competency.^8^ However, negative HCP interactions, including discrimination often resulting from homophobia,^12 13^ have been reported. A 2018 Stonewall report^14^ found that one in four LGBT people have witnessed discriminatory or negative remarks against LGBT people by healthcare staff. The same report found that one in seven LGBT people avoided seeking care due to fear of discrimination. This can be explained, in part, by minority stress theory, which posits that individuals who hold a minority identity are systematically stigmatised and feel prejudice and discrimination, which can create a hostile social environment.^12 15 16^ In contrast, a national US-based survey found that LGBTQ+ patients were six times more likely to be satisfied with their care when receiving treatment in an LGBTQ+ welcoming environment.^17^

For transgender (trans) patients, there are additional considerations^11 18^ which are compounded by small patient numbers and low levels of clinical oncology experience within trans health.^19^ We use ‘trans’ as an umbrella term for ‘identities, experiences and modes of gender presentation’ linked to a ‘social and/or physical transition’(p2).^20^ Other aspects of care can have emotional or psychological impacts; for example, misgendering by medical staff^21^ or gender-specific provision, such as single-sex hospital wards, including for those identifying as non-binary who do not exclusively identify with the binary categorisation of gender identity. In addition, treatment for hormone-related cancers can interfere with the administration of gender-affirming hormone therapy.^22^ These challenges reportedly discourage help-seeking behaviours^4 23^ and may contribute to trans patients being more likely to present with later stage cancer and have worse survival than cisgender patients .^24^

Uterine cancer (UC), which includes endometrial cancer (EC), is the most common gynaecological cancer in the UK.^25^ The incidence of UC in LGBTQ+ patients is unknown; however, a US-based study reported that EC risk may be higher in lesbians than their heterosexual sisters due to lower pregnancy and higher body mass index rates reported in the study sample.^26 27^ Suspected UC is typically investigated by transvaginal ultrasound scanning and hysteroscopic biopsy, often performed as an outpatient procedure with minimal analgesia and without anaesthesia.^28^ UC treatment is primarily surgical,^28^ with the majority of procedures performed by minimally invasive surgery, typically involving transvaginal extraction of the surgical specimen.^29^ In addition, pelvic and speculum examinations form the mainstay of clinical follow-up and can be associated with discomfort, fear and anxiety.^30 31^

The experiences of UC patients are under-represented in the literature,^32^ particularly within the qualitative research domain where often gynaecological cancers are grouped together, in particular with cervical cancer, thus limiting the specific knowledge that can be extracted to tailor future interventions.^33^ This is important when considering the variations in characteristics of different populations of gynaecological cancer patients such as age, ethnicity,^34^ cancer-related risk factors and aetiologies, and how such variations might impact experiences and needs.^35 36^ Findings derived from research conducted elsewhere geographically should also be applied with caution given the varying cultures, risk factors and healthcare organisation and practices which might underpin them.^37^ Given the increasing incidence of UC, the lack of public awareness in the UK, and the potential discrepancies in care for LGBTQ+ patients, understanding the experiences of LGBTQ+UC patients is essential to inform how healthcare can serve the specific needs of this population. Previous research has also identified gaps in HCP training, knowledge and perceived competency.^38^ Thus, establishing an understanding of how such needs are being (un)met and ways to move forward was also a priority of this exploration. The aim of this study was to develop a nuanced, qualitative understanding of current UC care for LGBTQ+ patients, incorporating multistakeholder perspectives from LGBTQ+ UC patients, patient partners and individuals who support UC patients including HCPs and cancer support charity staff.

## Methods

Ethical approval was granted by the University of Leicester Medicine and Biological Sciences Research Ethics Committee (reference 33691). This study has been reported in line with consolidated criteria for reporting qualitative research (COREQ) criteria for reporting of qualitative research^39^ (online supplemental file S1).

### Patient and public involvement

Patients from the target group were not involved in the initial design of the research due to time constraints. However, stakeholder insights were integrated into this study through working with a representative of an LGBTQ+ cancer charity. An online meeting between the lead researcher and the charity representative took place during the recruitment and data collection stage. The aim of this meeting was to discuss ways to better tailor recruitment strategies and to sense check findings to inform further refinement of interview schedules.

### Participants

Participants were recruited from three populations: (1) patients who had completed treatment for UC and who self-identified as LGBTQ+; (2) partners of LGBTQ+UC patients and (3) individuals who play a role in the provision of UC, including National Health Service (NHS) HCPs and those who provide advocacy and care for people affected by UC through cancer support charities.

### Recruitment

Purposive recruitment took place via social media, engagement with relevant cancer support charities and snowball sampling through word of mouth. Participant-facing documentation referred to UC as ‘womb cancer’, which is a more commonly used and understood lay-term. All participants were provided with a participant information sheet along with a verbal explanation via telephone call prior to providing informed consent. NM conducted all interviews with participants online using *Microsoft Teams* or via telephone

### Interview schedules

Semi-structured interview schedules for the participant cohorts were designed to explore subjective experience. Topic areas were informed by the research question, the research team’s clinical experience and previous qualitative research on the topic (e.g.^12^). Interview schedules for patients posed questions related to the meanings they assigned to cancer previous to their diagnosis, their experiences of the healthcare environment and survivorship. Specific questions and interview schedules for all cohorts are available in online supplemental file S2. The schedules were flexible to reflect the nuanced experiences and identities of participants, and participants were invited to elaborate on areas of importance to them. NM has PhD and postdoctoral-level experience of using this qualitative research method.

#### Positionality

Identities and experience of the four members of the research team varied. NM is a postdoctoral health psychology researcher, with experience of working with trans participant groups and in gynaecological cancer; EM is a clinical academic specialising in UC; RB is a social scientist and lesbian whose research expertise relates to LGBTQ+ health and domestic abuse and ND is a social scientist and Leicester NIHR Biomedical Research Centre Director of Inclusion, whose research expertise focuses on health inequalities. Prior to and during the research process, the research team engaged in reflexive practice, noting that their varying personal, vocational and research backgrounds provided a multidisciplinary approach to the development of interview questions and corresponding prompts.

### Data analysis

An inductive analytical approach was taken to Braun and Clarke’s guidelines on reflexive thematic analysis.^40 41^ The process of reflexivity on the part of the lead researcher began prior to data collection through self-questioning based on recent guidance by Braun and colleagues.^42^ Such questioning was done both explicitly and implicitly, alone and with other members of the research team. This enabled NM to identify inherent and recurring assumptions alongside placing knowledge in relation to previous experiences and hopes for the current study. On completion of data collection, audio recordings of interviews were transcribed verbatim using the *Microsoft Teams* transcription alongside manual checking for accuracy. All names were excluded from the data to ensure participant confidentiality and maintain anonymity. The data were coded initially by NM and then additionally a subset of the data was coded by both NM and RB. They met to compare codes and to apply reflexive practice in the process of sense checking assigned meaning. No outstanding differences in interpretation were identified and thus a consensus was reached between both researchers allowing for a credible and trustworthy characterisation of the data and its interpretation. An iterative data analysis process led to the identification of three main themes, with associated subthemes.

## Results

Fifteen participants took part in an interview. There were no instances of people declining to participate after they had been approached by the study team. The average length of interviews was 46 minutes. There were three patients in cohort 1 and one patient partner in cohort 2, with a mean age of 44 years (range 38 years to 56 years). Patients have been given pseudonyms of Lisa (cisgender lesbian), Emma (cisgender lesbian) and Sam, who identified as non-binary trans masculine, meaning someone who identifies more with the masculine side of the spectrum. Further demographic data were not collected to avoid compromising participant identity. In cohort 3, there were three cancer support charity staff, four gynaecologists/oncologists and four CNSs. Some HCPs and charity staff voluntarily disclosed their identities, with five HCPs and one charity staff member reported identifying as LGBTQ+. Geographical regions in which they worked are not reported to maintain participant anonymity

Three themes were identified during analysis: (1) identity disclosure; (2) not fitting in, and (3) righting the wrongs.

### Identity disclosure

The topic of disclosure was predominant across all interviews. This theme described how patients felt about disclosing their sexual orientation and gender identity (SOGI), and how these disclosure opportunities present themselves within current history-taking protocol.

#### The relevance of disclosure

In the context of UC care, a HCP is the first point of contact to disclose relevant personal information to initiate rapport building and inform decision-making. In particular, patients’ experience of diagnostic outpatient hysteroscopy procedures varied depending on disclosure opportunities and were reflected in interactions with clinicians, including inappropriate comments. Patients suggested that clinical consultations may have been improved if additional personal information had been sought prior to carrying out the procedure (online supplemental file table S3.1). Such experiences were felt to create a stigmatised environment during an already vulnerable time. Sam highlighted the relevance of gender identity disclosure in relation to undergoing a hysteroscopy, because of vaginal penetration being triggering for them: ‘I have never been interested in anything penetration wise, so that sort of thing was unpleasant, uncomfortable and quite frankly, a bit triggering’ (P10, cohort 1).

#### Disclosure opportunities

Opportunities to disclose personal information varied, and it was suggested that the way in which these opportunities presented themselves was inequitable. For Lisa and Emma, who both identified as cisgender lesbians, disclosure happened because their partner had accompanied them to the appointment and they felt comfortable to disclose the nature of their relationship: ‘I normally say I'm gay or my partner is here. You know, I've got to an age where I don't care. I don't even care if they care these days’ (P2, cohort 1). Emma’s wife reflected positively on her experience, suggesting that she felt welcomed by staff: ‘I didn't think it was a big deal. It’s not something that we've had to say constantly. I think the regular faces that we were seeing that knew about it were quite supportive’ (P6, cohort 2).

HCPs explained that asking patients who they have brought with them, along with enquiring about social support, are the recommended approaches to rapport-building. They suggested that effective relationship-building which promotes trust can provide opportunities for patients to disclose personal insights which they think are relevant to their care, rather than asking for specific information about sex and gender. This was echoed by a CNS who identified as queer:

I am not sure it should be (asked by HCP). If I’ve been told I have cancer of the womb, are you a lesbian? I’m not sure of the relevance. I want to know that my partner is going to be included in the decisions, I want to know that she can visit me the same as a husband, to know that all my questions are going to be answered, that there aren’t going to be any prejudices. I don’t know if I would need anything special other than that. (P1, cohort 3)

However, for Sam, who did not present with a partner, the onus was placed on them to inform clinicians about their identity. They found this challenging to navigate, particularly in the context of having limited appointment time and being faced with an internal examination (hysteroscopy):

It never really came up earlier on. I briefly mentioned it, but I certainly wasn't discussing it in any detail. I think if somebody had had more than just a brief meeting with me, I might have gone into more detail but it seemed more about just getting it over with. It (the hysteroscopy) might have been easier if I had actually explained fully and allowed them to understand why (P10, cohort 1).

Thus, in spite of all patients being provided with ostensibly *equal*, protocolled care, such care may not be *equitable* for LGBTQ+ patients, where the quality of their care may be adversely impacted by the lack of opportunity to disclose identities under a predominantly heteronormative care protocol.

### Not fitting in

A feeling of not fitting into the gynaecological cancer environment was perceived to be a particular challenge for patients, due to the cis-normative and heteronormative assumptions which often underpinned interactions. This theme details how participants appraised this aspect of UC care.

#### A gendered space

It was acknowledged that UC care and the associated language reflects a feminising and gendered space for patients, as exampled by a HCP: ‘The word Women’s Centre. It can feel very empowering for the women, but it can, for a very small number of people feel exclusionary and really at odds with where they are and where we are’ (P4, cohort 3). For Sam, who identified as trans masculine, a womb cancer diagnosis and its after-effects (eg, menopausal symptoms) were perceived to be a feminising experience, and this was heightened by the logistical arrangement of being in a female waiting room: ‘I was making a conscious attempt to be more feminine rather than masculine sitting in a room full of women’ (P10, cohort 1), and by interactions with others: ‘I did get some funny looks and it’s just like clearly I'm here for a reason. It’s none of your business, that was rough. Even the gynaecology consultants all seemed to think of me as somewhat of an oddity’ (P10, cohort 1).

Anecdotes of bias and prejudice towards trans patients by clinicians were reported by HCPs. For example, a CNS recounted an interaction that they had witnessed between a trans man and a gynaecologist:

The gynaecologist went to the waiting room to call her patient, and on seeing this gentleman said ‘I think you’re in the wrong place sir, where is it that you want to go to?’ And he said ‘gynaecology’ and she said ‘well I don’t think so, you’re a man. (P1, cohort 3)

A third sector worker who advocates for LGBTQ+ cancer patients commented that such a gendered environment may also be conflicting for cisgender women: ‘There’s quite a lot of queer women who also don't respond well to this hyper-gendered environment. Like, that’s something patients really push back against this idea that cancer is pink and fluffy because it’s just not, right?’ (P9, cohort 3). Hence, implicit gendering of a cancer such as UC can adversely impact patient experiences of the treatment pathway.

#### Assumptions-based care

The patient participants frequently referred to experiences within the UC pathway which were underpinned by cis-heteronormative assumptions, leading to feelings of marginalisation and neglect. This particularly focused on vaginal examinations and fertility wishes, with an assumption that LGBTQ+ patients would not want children and that therefore fertility preservation was not relevant. Sam expressed frustration at the negative impact of assumption-making more generally: ‘There is more than one type of female person that you could be dealing with, so don't assume it’s all the same and by choice, a lot of people will not visit a gynaecologist because of what they are’ (P10, cohort 1).

Information on treatment-related effects and UC survivorship was also perceived to be impacted by these implicit assumptions, most notably with reference to guidance on sexual function and sexual well-being after womb cancer. This is illustrated by Lisa’s account of the restoration of sexual function revolving exclusively around penile-vaginal intercourse:

The information about sex all assumed that it was going to be a penis. You’re supposed to use dilators after, so I have started doing that but they are totally heterosexual orientated, all the descriptions. It assumes sort of one way of having sex which is not even true in the heterosexual world. (P3, cohort 1)

This lack of adequate information was reiterated by HCPs:

I mean, if they (patients) can’t find it, it’s hard for us to find it. We’d be looking on the internet for what’s available, and we don’t have any local groups for the LGBTQ+ community that we can point them in that direction. (P15, cohort 3).

HCPs also referred to relying on assumption-making to know when to initiate conversation about sexual well-being, and how they can be inherently biased in their approach based on their own personal and previous experiences (online supplemental file table S3.2). These experiences suggest that the predominant assumptions among HCPs are underpinned by a heteronormative view of gynaecological health which may lead to specific needs of LGBTQ+patients remaining unmet.

### Righting the wrongs

Insights from clinicians alongside specific patient reflections suggest that there are a number of areas of care that require change in order for UC pathways to be more inclusive. The final theme examines participants’ perceptions of training and education for clinicians and how to instil best practice going forward.

#### Not just a tick box exercise

Clinicians unanimously reported that they had received no formal or structured training or education on the potential needs of LGBTQ+ patients (online supplemental files S3.3). If training was offered, it was reflected on as tokenistic and lacking meaningful and long-lasting change in its approach: ‘It’s much easier for us to tick boxes online than it is for us to do anything meaningful, and unfortunately, that is kind of what all of that training looks like now’ (P8, cohort 3).

Positively, a number of HCPs explained that they or their colleagues had been involved in the initiation of LGBTQ+ health-related modules in medical school to educate students. Whilst HCPs referred to occasional LGBTQ+ focused training days, these were typically frequented by clinicians with a personal interest or who identified as LGBTQ+ themselves: ‘I only really volunteered for it because I’m part of the community. I’d say eighty percent of the people there were from the LGBTQ+ community so I think it’s something that should be offered to all nurses, all doctors’ (P1, cohort 3).

A lack of education and training opportunities were also seen to feed into a ‘fear’ from clinicians regarding their use of language and own competency during interactions with patients. A similar concern was experienced by a charity representative who felt she lacked knowledge on how to represent the LGBTQ+ community appropriately and inclusively, such as, *‘*It could be perceived as just a box ticking and we don’t want to offend’ (P13, cohort 3). Thus, education which prioritises lived experience of LGBTQ+ patients and training which helps to embed inclusive language was suggested.

#### Contested agendas

Increased visibility of LGBTQ+patients within gynaecology was seen as paramount to overcome existing systemic barriers to equitable care provision, namely through recording demographics such as trans identity and sexual orientation, for example:

The NHS does a really poor job of monitoring sexual orientation and trans status in clinical record. They just don’t see it as a priority. So, what it leaves our community in is a catch twenty-two where we struggle to gain any sort of financial foothold or advancement or national programme for education because people say, well, there aren’t any numbers. (P9, cohort 3)

This lack of patient demographic monitoring to drive an equitable agenda was seen by this participant to represent systemic homophobia. However, for HCPs, the collection of such information was a contested topic, and many felt that this was unnecessary for patients to receive quality care. A CNS reflected that over-prioritisation of protocol questions may negatively impact rapport-building:

If it’s protocol, it sounds like a call centre, and I don't think that works because that’s not terribly individualised. It’s like ‘ohh I can't do this unless I have a script’. We've got to encourage the art of medicine and the development of effective communication skills. (P14, cohort 3).

Some HCPs also suggested that patients may not feel comfortable being asked to disclose such information and this may lead to further marginalisation. However, the patients who participated in this research predominantly wanted more opportunities to disclose their LGBTQ+ identity, to avoid treatment being affected by incorrect assumptions. As Lisa reflected:

I think the big gap is if you haven’t got somebody like me who’s quite confident in who they are, and you don’t tell people, I think a lot is assumed. So, it would be really good to try and build into some of the process, either some other information, particularly around sex, or some questions that are asked (P2, cohort 1)

This quote highlights a significant inequity in UC care provision between those who identify as LGBTQ+ and those who do not, as it often becomes the responsibility of the patient to educate the clinical team on their needs, which may be mediated by a patient’s confidence to do so.

## Discussion

This study’s findings uncover specific care needs of LGBTQ+ UC patients, whilst also highlighting the relevance of previous findings to this group of patients and care providers. The NHS’s cis-heteronormative approach was clearly demonstrated in this study, and the triangulated results point towards heightened marginalisation on the part of LGBTQ+ patients. This was in part due to the intersection of gynaecological cancer vulnerability^43^ and minority stress within the context of a care environment which is underpinned by cis-heteronormative practices. Thus, the participants in our study represent a patient group who may feel vulnerable due to their cancer diagnosis, and also perceive and/or experience additional stigma and discrimination due to their sexuality and gender identities. Previous research has linked the concept of felt and enacted stigma within a minority stress framework^15^ to opportunities for disclosure^12^, and the current study provides extended evidence for this within UC care.

HCPs in the UK have a duty of care to treat all people fairly and equally; however, *equal* care for UC patients may not result in *equitable* care that serves the additional needs of LGBTQ+ patients. This particularly pertains to the anxieties associated with pelvic examinations^30^ and inclusive survivorship resources. A circulating tumour DNA (ctDNA) blood test has been shown to be highly sensitive in detecting UC recurrence many months before clinical or radiological recurrence.^29^ The development of a ctDNA-led follow-up scheme could bring additional benefits for the LGBTQ+ population by enabling risk stratification of cases thereby avoiding the necessity of pelvic examination in patients with a negative ctDNA test. Survivorship information on sexual health and sexual function after UC was found to not be inclusive of, or relevant to, patients in our study, echoing the sparse literature on the sexual health of LGBTQ+ oncology patients .^44^ HCPs also commented on the challenges of providing information which was relevant and specific to the needs LGBTQ+ patients.

There were divergent opinions held by HCPs on the relevance of and process for collecting SOGI information with tensions between collection of such information via protocol questioning versus through personalised care and rapport building. Conversely, patients reflected on the pivotal role played by disclosure within gynaecological cancer, as they felt they were disproportionately impacted due to their LGBTQ+ identity and the assumptions associated with having a gendered and feminising cancer. There were also suggestions from patients that disclosure of LGBTQ+ identity may vary depending on one’s own confidence and feelings of comfort and trust in the gynaecological cancer environment.

The perspectives of a number of HCPs in our study on collecting SOGI data is supported by a study from UK GP practices,^45^ finding that staff tended to mirror their anxieties in their monitoring behaviour. UK-based medical students reported confidence to discuss sexual orientation during patient interactions increased with year of study, whereas confidence in discussing gender identity did not.^46^ Disclosure during cancer treatment is associated with higher levels of psychological well-being and mental health, comfort and increased satisfaction for patients,^47^ therefore a balance of SOGI data collection and creation a of safe environment for this disclosure is needed. Our findings highlight a call for HCP education on the diverse and additional needs of LGBTQ+ patients, while previous research highlights HCPs’ desire for specific training.^48^ Three out of four UK-based HCPs have reported wanting education on LGBTQ+ health in their training,^48^ therefore such efforts could be an effective and engaging avenue for creating an inclusive UC environment for patients. However, key findings from this study highlight the need for such education to instil meaningful change to promote personalised interaction, rather than a homogenising or tokenistic approach which might further marginalise or stigmatise LGBTQ+ patients.

### Methodological considerations and future directions

In order to address the invisibility of LGBTQ+ patients in the UC literature, we analysed the current data set through a lens of prioritising patient accounts and using HCP and stakeholder experiences to provide contextual understanding. Despite the patient group in this study including both lesbian women and trans people, the small sample size of patients is a limitation, particularly given that we chose to approach the research question by combining sexual and gender identities, which can conflate findings. To mitigate this, where possible, we specified whether a finding was specific to sexual orientation or trans identity, or both. We are cautious to note that while we have collected rich data from some LGBTQ+ patients, we make no claims to represent the wider population of LGBTQ+ patients. This is important to emphasise, given that LGBTQ+ people are a very heterogeneous group.

The decision was made not to recruit patients through the NHS and their usual care team since it was felt that individuals may be reluctant to share negative experiences because of concerns of an impact on their ongoing clinical care, as well as disclosing their LGBTQ+ identity more widely. Additionally, given that SOGI information is not collected on hospital databases, there would have been no systematic or efficient way of recruiting LGBTQ+ patients via NHS channels. We would suggest that future researchers work closely with a patient and public involvement group to optimise recruitment and set research priorities, and to carefully consider recruitment materials and lengthy timescales for recruitment during study design.

Due to considerations about participants being identifiable, we collected only patient age and not demographic indicators (eg, geographical location and ethnicity) within this study. Given that we identified an intersecting role of minority stress in the gynaecological cancer environment, we would hypothesise that there are additional minoritised identities such as race, ethnicity, socioeconomic status and educational attainment which may influence patient experience and interactions with HCPs. It would be valuable for future research to prioritise the recruitment of a larger, diverse sample to further our understanding of how these cancer care pathway-related processes disproportionately affect different groups of LGBTQ+ patients.

## Conclusion

LGBTQ+ UC patients have specific care needs and considerations, particularly related to transvaginal procedures and survivorship. Opportunities to disclose LGBTQ+ identity during the diagnostic pathway were considered important for these needs to be recognised. Despite this, there are conflicting agendas between HCPs and stakeholders on the best approach to integrate disclosure processes. The current findings highlight how cis-heteronormative protocolled care can lead to stigmatising experiences for LGBTQ+ patients. The need for meaningful and applicable education for healthcare providers on how to approach UC care to establish trust and to create a safe environment for LGBTQ+ patients is paramount.

## supplementary material

10.1136/bmjopen-2024-084720 online supplemental file 1

10.1136/bmjopen-2024-084720 online supplemental file 2

10.1136/bmjopen-2024-084720 online supplemental file 3

## Data Availability

No data are available.
