# Supplementary material for: Qualitative exploration of uterine cancer care for lesbian, gay, bisexual, trans and queer (LGBTQ+) patients in the UK: shifting from equality to equity
Source: BMJ Open. 2024 Aug 2;14(8):e084720. doi: 10.1136/bmjopen-2024-084720 (PMC11298749; doi:10.1136/bmjopen-2024-084720)
Supplement: online supplemental file 2 [file bmjopen-14-8-s002.pdf]

Supplementary material S2

S2 - Interview schedules

S2.1. Semi-structured interview (patients and partners)

**Topic**	**Patient questions**	**Partner questions**
Prior awareness and perceptions of womb cancer	• Prior to your diagnosis, when you thought of cancer, what came to your mind?<br><br>• Can you tell me whether you had heard of womb cancer before your diagnosis?<br>- Why do you think that was?<br>- Media<br>- Organisations<br><br>• Did you know anyone else who had been diagnosed with womb cancer?<br>- Who<br>- Treatment<br>- Relationship with person<br><br>• How do you think this prior knowledge of/ lack of knowledge of womb cancer impacted your diagnosis experience?<br><br>• Were you aware of any campaigns which raised awareness of womb cancer?<br>• Appropriateness/ sensitivity of such campaigns<br><br>• How do you think womb cancer is perceived within the LGBT+ community?	• Had you heard of womb cancer prior to your partner's diagnosis?<br><br>• Were you aware of any attitudes towards womb cancer within the LGBT+ community?<br><br>• Did you know of anyone who had been diagnosed prior to your partner?<br>- Who<br>- Relationship<br><br>• Do you think your knowledge/ lack of knowledge of womb cancer impacted your experience?
Symptoms and diagnosis	• What were the symptoms that you had which led you to seeing your GP/ doctor?<br>- Awareness of symptoms linked to womb cancer?	• Can you tell me about how your partner was diagnosed with womb cancer?<br><br>• How did your partner's diagnosis make you feel?

	- What did you think was causing these symptoms? Why did you think this?<br><br>• Were you aware of any factors that might increase your risk of getting womb cancer?<br><br>• What do you think of surgeries which can reduce the likeliness of womb cancer developing?	- Why do you think you felt that way?
Interactions with HCPs; hospital and treatment environment	• Can you tell me about how you found your interactions with your doctor?<br>- Disclosure of sexuality?<br>- Sensitivity to disclosure<br>- Trust/ safety<br><br>• Can you tell me about the treatment that you had for womb cancer?<br><br>• How did you feel about the environment that you were being treated in, for example, did you feel safe or welcome?<br>- Any specific environmental cues for LGBT+ support<br><br>• Is there anything that you would change within the hospital environment to make it more welcoming for LGBT+ people?	• How was your initial interaction with your partner's doctor?<br><br>• How did you perceive this doctor's knowledge and attitude towards you and your partner's relationship?<br><br>• How well equipped was this doctor to provide relevant information for you and your partner?<br><br>• Was this information tailored to your LGBT+ relationship?<br>- Can you provide an example of this?<br><br>• How do you think this impacted your experience?<br><br>• Can you tell me about how it was for you when your partner was receiving treatment?<br><br>• What role did you play during this time?<br><br>• Why do you think you took on this role?<br><br>• How did you feel about this?<br><br>• What were the main challenges that you experienced during your

Supplementary material S2

		partner's treatment? (Ask for specific examples)<br><br>• Was there anything that you found particularly helpful during this time?<br><br>• How were your interactions with your partner's doctor during treatment?<br><br>• Did you receive adequate information from them?<br><br>• Do you think that you received support during this time?<br><br>• Did you feel accepted in the hospital environment?
Survivorship	• How did you find the time period after finishing treatment?<br>- Any challenges?<br><br>• What were your experiences of clinical follow-ups e.g., pelvic examinations? (if not already mentioned)<br><br>• Can you tell me about your experiences with/ attitudes towards sex since having treatment?<br><br>• Have you noticed any differences in your sexual function?<br>- Impact of hysterectomy<br>- Impact of menopause<br><br>• Do you think your gender identity has been impacted by treatment?<br><br>• Can you tell me about anything that could have improved your experience since treatment?	• How has it been for you since your partner finished treatment?<br>- Psychologically<br>- Socially<br><br>• Have you noticed any changes in your relationship since your partner's treatment?<br>- Physically<br>- Sexually<br>- Socially<br><br>• What have been the main challenges for you since your partner finished treatment?<br><br>• How has your everyday life been impacted?<br>- Caring responsibilities<br>- Work<br>- Priorities<br><br>• Have there been any worries since your partner's treatment?<br>- Did you receive support/ information for these worries?<br><br>• What has helped you since your partner's treatment?

	- Physical<br>- Psychological<br>- Social<br>- From an LGBT+ perspective<br><br>• Do you feel as though you have been offered adequate information or resources to help with your needs since treatment?<br><br>• Do you think there are any ways that the experience of LGBT+ patients could be improved in the time period after womb cancer?<br><br>• Is there anything else that you would like to say about your experience of diagnosis, treatment or follow-up/ survivorship?	• What advice would you give to someone else whose partner is going through womb cancer?<br><br>• Is there anything else that you would like to say about your experience?

Supplementary material S2

S2.2. Semi- structured interview schedule (Clinicians and organisation representatives)

Demographic information

1. Firstly, I am just going to ask some questions for our records, but just to emphasise that all personal information is stored confidentially and you won't be identifiable from this. So, can you start off by telling me….

- What your occupation is? How long have you been in this position/ role?
- The geographical location of the clinic/ organisation that you work in?

Interaction with patients

For third sector organisations only

2. Can you tell me about the organisation that you work for?
   - How long has it been running?
   - What are its main goals?
   - What are the main services that it provides?

3. Can you tell me about what care or support you provide to patients?
4. What are the main roles involved in your position?

Experience of providing care to LGBT+ patients

5. What has your experience been of providing care to patients who identify as LGBT+?; Have you ever, to your knowledge, provided care to a patient who identified as LGBT+

   If yes:

   - How did you know that they identified as LGBT+ ?; Did they disclose the information to you?
   - Did their being LGBT+ affect the information that you provided to them or any decisions that were made about their care? Prompt next question?

     For clinicians only:

   - Did the disclosure impact how you approached pelvic examination of the patient/ or whether you would consider non-invasive treatments?

   If no:

- Do you think it is important for LGBT+ patients to feel as though they can disclose their gender identity and/or sexual orientation? Why do you think this?
- What things would you consider if a patient disclosed that they identified as LGBT+?
- PROMPT: Are there any specific needs that LGBT+ patients might have?

6. What do you think the potential challenges for LGBT+ patients in a gynecology setting?
   - Are there particular conversations/ observations that have informed this?
   - Are there particular assessments or treatments options that you think might be particularly challenging? Why do you think this?
   - What are the potential challenges are for LGBT+ patients during the time period after cancer?

Knowledge

7. How do you perceive your own knowledge of LGBT+ patients' needs?
8. Where did you think this knowledge has come from?
9. Can you tell me about any education or training that you have received in providing care for LGBT+ patients?
   - How was this delivered?
   - What were the key take home messages?
   - Were there any gaps in this training?
10. Do you think training / education such as this is needed? If so, how would it help you in your role?

Recommendations

11. What do you think could be changed to provide cancer care for LGBT+ patients?
12. Is there anything else that you would like to add on this topic?
