# Supplementary material for: Qualitative exploration of uterine cancer care for lesbian, gay, bisexual, trans and queer (LGBTQ+) patients in the UK: shifting from equality to equity
Source: BMJ Open. 2024 Aug 2;14(8):e084720. doi: 10.1136/bmjopen-2024-084720 (PMC11298749; doi:10.1136/bmjopen-2024-084720)
Supplement: online supplemental file 3 [file bmjopen-14-8-s003.pdf]

Supplementary material S3

S3- Participant quotes

Table S3.1. Quotes illustrating the relevance of disclosure within uterine cancer care

Negative interaction with clinician	In fact, my cervix was closed. I thought the clinician was a bit annoyed about that. Yes, she was, just didn't react. Just said, this is going to hurt. So that was the one bit I thought actually they hadn't really taken into account anything about me. I haven't had children (P2, Cohort 1).
Negative interaction with clinician	The consultant made a couple of passing comments about, well, ‘do you not even use toys?’ It's like, no, no, that's no. It wasn't great” (P10, cohort 1).
Inappropriate protocol questions	But it's the whole ‘are you going to be pregnant?’ well not only am I going to go for a hysterectomy, I'm a lesbian as well’ So you kind of feel like you're repeating yourself a little bit. (P3, Cohort 1)
Positive impact of disclosure	I felt more comfortable probably disclosing it to people at the hospital than I would just in general (P8, cohort 2)

Cohort 1= Patients who had completed treatment for UC and who self-identified as LGBTQ+; Cohort 2= Partners of UC patients; Cohort 3= Individuals who play a role in the provision of uterine cancer those who provide advocacy and care for people affected by UC

Table S3.2. Quotes illustrating experiences of assumptions-based care

Assumption about women being used to vaginal examinations	It was an assumption that you were used to being examined and people look into your vagina and for me it was all sort of the first time really apart from smear tests (P2, cohort 1)
Prioritisation of fertility	If it's not a fertility issue because you don't want kids, then it's just 'well you need to deal with it [symptoms]'. It took me until I was in my thirties before someone even recommended that I go on the pill to sort my periods out (P10, cohort 1)
Assumption about homosexual couples not wanting children	One of the pitfalls is perhaps people don't have the same conversation as they would with a straight couple and perhaps one of the assumptions is that they are not going to have children" (P4, cohort 3)
Assumptions around discussing sexual health	Even I know that you shouldn't really make a judgment call because how do I know that this person sitting in front of me, because I'm thinking, oh, they're seventy, they don't want to discuss these things" (P5, cohort 3)

Cohort 1= Patients who had completed treatment for UC and who self-identified as LGBTQ+; Cohort 3= Individuals who play a role in the provision of uterine cancer those who provide advocacy and care for people affected by UC

Table S3.3. Quotes reflecting state of training and education for healthcare professionals on the needs of patients

Lack of training and education opportunities	In Med school, I wasn't taught about LGBT health" (P5, cohort 3)
Lack of training and education opportunities	It wasn't really something that was talked about much when I was at medical school (P12, cohort 3).
Specific training not prioritised	I think at the same time there was a stream on robotic surgery. So I went to that instead. It was the first time I was aware of any LGBTQ plus teaching at any conference or anything (P6, cohort 3).

Cohort 3= Individuals who play a role in the provision of uterine cancer those who provide advocacy and care for people affected by UC
