# Supplementary material for: Qualitative exploration of uterine cancer care for lesbian, gay, bisexual, trans and queer (LGBTQ+) patients in the UK: shifting from equality to equity
Source: BMJ Open. 2024 Aug 2;14(8):e084720. doi: 10.1136/bmjopen-2024-084720 (PMC11298749; doi:10.1136/bmjopen-2024-084720)
Supplement: Supplementary file 4 [file bmjopen-2024-084720.reviewer_comments.pdf]

PEER REVIEW HISTORY

BMJ Open publishes all reviews undertaken for accepted manuscripts. Reviewers are asked to complete a checklist review form (**http://bmjopen.bmj.com/site/about/resources/checklist.pdf**) and are provided with free text boxes to elaborate on their assessment. These free text comments are reproduced below.

ARTICLE DETAILS

**TITLE (PROVISIONAL)**	A Qualitative Exploration of Uterine Cancer Care for Lesbian, Gay, Bisexual, Trans, and Queer (LGBTQ+) Patients in the UK: Shifting from Equality to Equity.
**AUTHORS**	Millet, Nessa; Barnes, Rebecca; Darko, Natalie; Moss, Esther

VERSION 1 – REVIEW

**REVIEWER**	Wearn, Angela<br>Newcastle University Faculty of Medical Sciences, Population Health Sciences Institute
**REVIEW RETURNED**	16-Feb-2024

**GENERAL COMMENTS**	I enjoyed reviewing this interesting and well written paper. There is no avoiding the main limitation that you have such a small number of patients (n=3) in your participant sample, when your paper focuses on patient experience of care. However, I thought there were valuable points to consider arising from your study including a strong conclusion which summarised the key points well. Despite this, there are some issues I feel should be addressed before the paper can be recommended for publication:<br><br>- The introduction section was well written and informative although I found the rationale for the study slightly lacking. For example, why is it important to triangulate the experiences of the groups you mention? How will doing so fill a 'gap' in current knowledge and/or help to inform care. Being specific about these points and your research question will help to clarify your reasoning for carrying out this particular piece of research.<br><br>- It was quite disappointing to read that there was no public involvement within this study which was so heavily focused on a patient experience (and particularly given this is a typically marginalised community). I would consider this as a limitation which you did seem to touch upon - but I felt the importance of addressing this could be emphasised a little more and considered a priority for future related work?<br><br>- Please provide more detail on the methodological and data collection approaches within the method section. Could the topic guides be included within the supplementary materials perhaps?<br><br>- Pg6, line 58 - Can you provide citations of the research you are referring to?<br><br>- Please clarify whether you intentionally did not collect demographic data or whether you did not report this due to the small sample size. Even an indication of age range and/or ethnicity

	would give insight into your findings given these things can also impact on care/stigmatisation etc.<br><br>-I'm wondering if an overview of the whole sample in terms of LGBTQ+ identity would be possible/ of value here - I am trying to get a sense of how well the sample can speak to the experiences of the community given there is such a small number of patient participants.<br><br>- It wasn't entirely clear why some quotes were presented in tables and some in text – I think the results section would benefit from a more consistent approach i.e. presenting quotes in either summary tables or in text.<br><br>- Pg 14, line 42-43 - Could you word this more clearly regarding how you have come to that interpretation from the data you are reporting? It seems incongruent with the previous sentence where you say most of the patients you spoke to had positive experiences.<br><br>- I think you could slightly expand on your point about the need to balance patients' personal decisions around disclosure with SOGI data collection. This for me is a really key aspect of the findings and also aligns with some of the quotes in the findings section regarding 'women's cancer care' being empowering for some and not for others. i.e. How do we move forward in making sure we balance the needs and preferences of multiple marginalised groups and avoid unintentionally worsening existing inequities?

**REVIEWER**	Ginsburg, Ophira<br>NCI, Center for Global Health
**REVIEW RETURNED**	19-Feb-2024

**GENERAL COMMENTS**	This is a nicely written and timely manuscript about the complex challenges faced by people with uterine cancer who identify as of diverse SOGI or LGBTQ+. I have several questions regarding the methods, including the small sample of patient participants, the rationale to focus on only people with uterine cancer, and the somewhat sparse literature review, given the considerable attention to the broader issue of gender and SOGIE-responsive health systems including for people experiencing cancer.<br><br>Major<br><br>1. It is unclear to me what this study adds to the growing literature on the challenges, needs, and potential solutions (including LGBTQ+ responsive cancer health systems). The literature reviewed does not include some key recent publications that would help to contextualize the study findings. I would encourage the authors to include (at least) some of the related literature from the US, e.g. the LGBTQ Cancer Network has published a major (recent) National survey, and a sub-analysis on trans populations with cancer, "gender expansive" findings and recommendations https://cancer-network.org/out-the-national-cancer-survey/<br><br>2. With only three participants having lived experience of uterine cancer, it seems not sufficient to warrant the conclusions. Can the researchers increase the sample with even a few more individuals with lived experience? The fact that one of the three (patient) participants identifies as trans makes it difficult to consider the

	unique challenges of people with uterine cancer from this population, which (other) literature suggests are often distinct from the cis-gender patient population.<br><br>3. Finally – the justification for focusing only on people with uterine cancer (vs other gynecological cancers) is unclear. There's substantial literature with relevant considerations for this population concerning other gynecological cancers, notably cervical cancer. If it was a problem of sampling, I would suggest extending to the population with cervical cancer as there are similar issues regarding diagnostic interventions, stigma, and other factors that might be highly salient to this exploration of challenges and potential solutions to achieving equity for cancer care in this diverse medically underserved population.

VERSION 1 – AUTHOR RESPONSE

**Reviewer 1 comments**	
The introduction section was well written and informative although I found the rationale for the study slightly lacking. For example, why is it important to triangulate the experiences of the groups you mention? How will doing so fill a 'gap' in current knowledge and/or help to inform care. Being specific about these points and your research question will help to clarify your reasoning for carrying out this particular piece of research.	Thank you for prompting us to provide a stronger rationale for conducting this study. We have included an additional paragraph which outlines the specific need for research amongst uterine cancer patients alongside that which also considers the experiences of HCPs in order to provide understanding of how such needs are (un)met and ways forward from a clinical perspective. (pages 5, line 22 – page 6, lines 1-14)

It was quite disappointing to read that there was no public involvement within this study which was so heavily focused on a patient experience (and particularly given this is a typically marginalised community). I would consider this as a limitation which you did seem to touch upon - but I felt the importance of addressing this could be emphasised a little more and considered a priority for future related work?	Thank you for noting this. We agree that PPI who represent patients would have strengthened this study's approach. However, logistical considerations of recruitment and time constraints limited our scope to be able to include such PPI. These considerations are also a reflection of one of the main findings in the study related to sexual orientation and gender identity data collection in clinic which increases the invisibility LGBTQ+ patients both in clinic and in research. A section has been added to the Strengths and limitations section at the start of the article (Page 3, line 10-12)<br><br>We did consult with a cancer and LGBT specific charity for advice on framing the study and on recruitment. We also then disseminated the study results back to the charity and other medical professionals. We have included this involvement in new section regarding PPI on Page 6 (Lines 10-15). Thank you for prompting us to recognise and include this in the write up of the study.
Please provide more detail on the methodological and data collection approaches within the method section. Could the topic guides be included within the supplementary materials perhaps?	Thank you for requesting more information, which we have included on Page 7, lines 19-24. Along with including the interview schedules in the supplementary materials (File S3). We have also provided further information on the reflexive approach to data analysis on: Page 8, line 13- page 9, line 1.
Pg6, line 58 - Can you provide citations of the research you are referring to?	Thank you for noting this. Citations have been added (Page 7, line 19)

Please clarify whether you intentionally did not collect demographic data or whether you did not report this due to the small sample size. Even an indication of age range and/or ethnicity would give insight into your findings given these things can also impact on care/stigmatisation etc.	Thank you for this suggestion. LGBTQ+ identity and age was only purposefully collected from patients. We did not collect other socio demographic data from patients given the small number of participants and the concern that they could be identified from their demographics (Page 9, lines 11-12).<br><br>HCPs and charity workers (cohort 3) were asked their role and the geographical location in which they worked. Given that a number of cohort 3 participants volunteered to disclose their identity, we decided to not also report the geographical location in which they work due to considerations of them being identifiable by this. Other identity related information was not collected as we felt this may deter participation.<br><br>As mentioned, a number of HCPs and charity workers shared their LGBTQ+ identity voluntarily at their own discretion. This has been clarified in paper (Page 9, lines 13-15)
I'm wondering if an overview of the whole sample in terms of LGBTQ+ identity would be possible/ of value here - I am trying to get a sense of how well the sample can speak to the experiences of the community given there is such a small number of patient participants.	Thank you for this suggestion. As mentioned, we did not collect LGBTQ+ identity for all participants. We have however taken your comment on board and have now included a more comprehensive overview of participant characteristics by also including age range of participants (Page 9, line 8)<br><br>The patient participants included two patients who self-identified as cisgender lesbian and one patient who identified as non-binary transmasculine. Whilst rich and valuable insights are gained from these LGBTQ+ patients, the size of this sub-sample and corresponding lack of diversity of a wider range of LGBTQ+ identities means that no claims to generalisability to LGBTQ+UC patients more broadly are made (Page 19, lines 4-7).
It wasn't entirely clear why some quotes were presented in tables and some in text – I think the results section would benefit from a more	Thank you for querying the approach we took to presenting the data.

consistent approach i.e. presenting quotes in either summary tables or in text	To make the paper accessible to a broad readership (clinical, academic and lay person), we decided to succinctly refer to the focal points of the findings using the most significant and more poignant quotes, with additional illustrative quotes in tables. We were inspired to use this approach on reading a similar approach in a relevant paper:<br><br>“It’s probably an STI because you’re gay”: a qualitative study of diagnostic error experiences in sexual and gender minority individuals | BMJ Quality & Safety<br><br>By taking this approach, we also acknowledge that triangulation of multiple perspectives may lead to imbalances in representation of experiences, particularly when reducing experiences down to a results section. Thus, to allow greater insight into lived experiences of the multiple participant groups, and to enable the reader to understand the weight behind certain points, we decided it was relevant to include extra data in tables. However, given that this presentation is not consistent, we have moved the tables to the supplementary material (Supplementary file 3).
Pg 14, line 42-43 - Could you word this more clearly regarding how you have come to that interpretation from the data you are reporting? It seems incongruent with the previous sentence where you say most of the patients you spoke to had positive experiences.	Thank you for noting this inconsistency. On reflection, we feel that this theme is largely informed by insights from clinicians. We included the contrast of ‘overall’ patient experiences to provide context but it is somewhat misleading. Thus, we have removed the first sentence and refocused the new leading sentence (page 14, lines 11-12)
I think you could slightly expand on your point about the need to balance patients’ personal decisions around disclosure with SOGI data collection. This for me is a really key aspect of the findings and also aligns with some of the quotes in the findings section regarding ‘women’s cancer care’ being empowering for some and not for others. i.e. How do we move forward in making sure we balance the needs and preferences of multiple marginalised groups and avoid unintentionally worsening existing inequities?	Thank you for making this very important point. We understand that there are different needs and concerns across the LGBTQ+ patient population. The data suggests that interactions which are both personalized and allow for open communication are paramount to enabling open disclosure at a level and time point that is helpful for the patient which is a key finding. In response to this suggestion, we have added a final sentence in the discussion to further distinguish between education which is meaningful and personalised compared to tokenistic or homogenising approaches. (Page 18, line 17-20)

Reviewer 2	
It is unclear to me what this study adds to the growing literature on the challenges, needs, and potential solutions (including LGBTQ+ responsive cancer health systems). The literature reviewed does not include some key recent publications that would help to contextualize the study findings. I would encourage the authors to include (at least) some of the related literature from the US, e.g. the LGBTQ Cancer Network has published a major (recent) National survey, and a sub-analysis on trans populations with cancer, "gender expansive" findings and recommendations https://cancer-network.org/out-the-national-cancer-survey/	Thank you for this important comment and prompt for us to further situate the need of this study and how it adds to the international literature base. Some additional literature, including the extensive survey that you directed us to, has been included in the introduction (Page 4, lines 17-24). However, we are mindful to not compare the UK healthcare environment and related needs of patients to that of other countries where cultures, risk factors and healthcare organisation and practices differ (Berner & Meads, 2022)<br><br>In addition, we have included an additional paragraph at the end of the introduction to establish what this study adds to the literature base (Page 5- page 6)
With only three participants having lived experience of uterine cancer, it seems not sufficient to warrant the conclusions. Can the researchers increase the sample with even a few more individuals with lived experience? The fact that one of the three (patient) participants identifies as trans makes it difficult to consider the unique challenges of people with uterine cancer from this population, which (other) literature suggests are often distinct from the cis-gender patient population.	Thank you for your suggestion. We agree that including 3 patients in the sample is indeed a limiting factor in terms of speaking to a broader set of patients with diverse lived experience. However, the aim of this paper was to examine UC cancer care experiences from multiple perspectives, not just patient experiences. Unfortunately, the period of funding for this study is over, and therefore we cannot add any more participants to the study, although we understand that if possible this would strengthen the findings.<br><br>It is worth noting that we recruited for this study for six months and in that time, only 3 patients came forward to be interviewed. This was despite us working with a Cancer and LGBT specific charity who gave advice on recruitment. Perhaps if SOGI information was routinely collected by NHS healthcare services then this recruitment period could have been more efficient. Thus, the small patient sample is in some ways a reflection of the issues found by this study, and a further reasoning for why despite a small number of patients, such patient voices are necessary to elevate and learn from to move forward. These considerations have been included in the strengths and limitations section (Page 3, lines 7-9)

Finally – the justification for focusing only on people with uterine cancer (vs other gynecological cancers) is unclear. There's substantial literature with relevant considerations for this population concerning other gynecological cancers, notably cervical cancer. If it was a problem of sampling, I would suggest extending to the population with cervical cancer as there are similar issues regarding diagnostic interventions, stigma, and other factors that might be highly salient to this exploration of challenges and potential solutions to achieving equity for cancer care in this diverse medically underserved population.	Thank you for this useful comment, which has prompted us to include additional information in the manuscript to explain why we have focused on uterine cancer patients (Page 5- page 6).<br><br>We acknowledge that grouping gynaecological cancers is the predominant approach given the similar diagnostic and treatment pathways. However, there are significant differences in the patient characteristics (e.g., age), aetiologies and risk factors associated with uterine cancer versus cervical cancer for example. Such differences impact how patients feel about their cancer and the needs that they have. whilst the literature base on cervical cancer is relatively strong, perhaps due to the existence of a screening programme, that for uterine / endometrial cancer is significantly lacking, with uterine cancer referred to as under represented both in funding and research (Wan et al., 2016). This is also reflected in the large scale US based survey that you referred to which does not include any endometrial cancer patients.

The new word count excluding references is **4,722 words**. This increase in word count is a result of changes made to respond to reviewer comments which we believe have significantly strengthened the paper through developing a stronger rationale and clarification of method and key findings.	

References

Berner, A. M., & Meads, C. (2022). LGBT Populations and Cancer in the UK. In U. Boehmer & G. Dennert (Eds.), *LGBT Populations and Cancer in the Global Context* (pp. 215-257). Springer International Publishing. https://doi.org/10.1007/978-3-031-06585-9_12

Wan, Y. L., Beverley-Stevenson, R., Carlisle, D., Clarke, S., Edmondson, R. J., Glover, S., Holland, J., Hughes, C., Kitchener, H. C., Kitson, S., Miles, T., Morley, R., Morrison, J., Nelson, L., Powell, M., Sadler, L., Tomlinson, A., Tylko-Hill, K., Whitcombe, J., & Crosbie, E. J. (2016). Working together to shape the endometrial cancer research agenda: The top ten unanswered research questions. *Gynecologic Oncology*, *143*(2), 287-293. https://doi.org/10.1016/j.ygyno.2016.08.333

VERSION 2 – REVIEW

**REVIEWER**	Wearn, Angela<br>Newcastle University Faculty of Medical Sciences, Population Health Sciences Institute
**REVIEW RETURNED**	10-May-2024

**GENERAL COMMENTS**	Thank you for your detailed response to the reviewer comments. I am satisfied that these have, overall, been addressed sufficiently. However there is one minor point that I feel should be clarified regarding patient and public involvement:

	i.e. Pg6, line 20 - It would be more transparent to say that you did not involve individual patients (i.e. as partners) within your research design and process (for example due to financial/time constraints) but worked with a representative of an LGBT cancer charity. Whilst I can understand why you have mentioned it within the paragraph, I also think it important to highlight that disseminating findings to a group is not public involvement activity.

VERSION 2 – AUTHOR RESPONSE

**Reviewer 1 comments**	
Pg6, line 20 - It would be more transparent to say that you did not involve individual patients (i.e. as partners) within your research design and process (for example due to financial/time constraints) but worked with a representative of an LGBT cancer charity. Whilst I can understand why you have mentioned it within the paragraph, I also think it important to highlight that disseminating findings to a group is not public involvement activity.	Thank you for prompting greater transparency in our reporting of PPI involvement. In response to this, we have changed this in the manuscript (Page 6, line 20-22).
